# Detecting and Predicting Pilot Mental Workload Using Heart Rate Variability: A Systematic Review

**DOI:** 10.3390/s24123723

**Published:** 2024-06-07

**Authors:** Peizheng Wang, Robert Houghton, Arnab Majumdar

**Affiliations:** Centre for Transport Studies, Department of Civil and Environmental Engineering, Imperial College London, London SW7 2AZ, UK; pw220@ic.ac.uk (P.W.); r.houghton18@imperial.ac.uk (R.H.)

**Keywords:** mental workload, ECG, heart rate variability, physiological signals, wearable devices

## Abstract

Measuring pilot mental workload (MWL) is crucial for enhancing aviation safety. However, MWL is a multi-dimensional construct that could be affected by multiple factors. Particularly, in the context of a more automated cockpit setting, the traditional methods of assessing pilot MWL may face challenges. Heart rate variability (HRV) has emerged as a potential tool for detecting pilot MWL during real-flight operations. This review aims to investigate the relationship between HRV and pilot MWL and to assess the performance of machine-learning-based MWL detection systems using HRV parameters. A total of 29 relevant papers were extracted from three databases for review based on rigorous eligibility criteria. We observed significant variability across the reviewed studies, including study designs and measurement methods, as well as machine-learning techniques. Inconsistent results were observed regarding the differences in HRV measures between pilots under varying levels of MWL. Furthermore, for studies that developed HRV-based MWL detection systems, we examined the diverse model settings and discovered that several advanced techniques could be used to address specific challenges. This review serves as a practical guide for researchers and practitioners who are interested in employing HRV indicators for evaluating MWL and wish to incorporate cutting-edge techniques into their MWL measurement approaches.

## 1. Introduction

Mental workload (MWL) is a key concern for all safety-critical industries as elevated levels of MWL can impair human performance, potentially leading to fatal accidents [1,2,3]. Conversely, extremely low MWL due to low arousal levels can cause boredom and lack of attention, also jeopardizing operational safety [4]. Aircraft piloting operations are typically complex sociotechnical systems, demanding the processing of diverse information from various sources, including visual and auditory cues, along with environmental inputs both within and outside the aircraft [5]. Thus, piloting an aircraft demands a high level of information processing and mental effort. The inability of pilots to effectively manage excessive MWL can jeopardize safety and operational efficacy, potentially resulting in catastrophic outcomes [6].

Safety statistics indicate that human errors, which are primarily related to aberrant MWL levels, contribute to approximately 70% of aircraft accidents [7]. A real-world example is the Turkish Airlines Flight TK1951 crash during its approach and landing, which tragically resulted in 9 fatalities, 120 injuries, and severe cockpit damage. The subsequent accident investigation revealed that a malfunctioning radio altimeter automatically activated the auto throttle, and the pilot failed to realize this due to an elevated MWL. Moreover, modern aircraft cockpits incorporate varying levels of automated systems, which could have a significant effect on pilot MWL. Lower-level automation can potentially elevate MWL as pilots may need to engage in various basic operations, while higher-level automation could diminish pilot situational awareness (SA), potentially resulting in “mental underload” [8]. In these contexts, the integration of accurate MWL measurement and prediction systems within the contemporary cockpit plays a crucial role in enhancing safety and proactively, mitigating aviation accidents [5]. This multi-faceted issue suggests the critical importance of ongoing research and technological development in effectively measuring and managing pilot MWL.

Despite the intuitive appeal of the concept of MWL across numerous domains, the lack of standardized terminology remains a persistent issue in the literature [9,10]. It is widely acknowledged that MWL is a multidimensional construct influenced by task demands, individual characteristics, and the surrounding environment. For the purpose of this review, we have adopted the following definition of MWL, which is relatively comprehensive and covers the terms previously mentioned: “the level of attentional resources required to meet both objective and subjective performance criteria, which may be mediated by task demands, external support, and past experience” [11]. It is important to note that although this review does not aim to establish a new definition of MWL, there exists a critical need to distinguish between taskload and MWL. Taskload is a highly task-dependent concept that can be simply defined as the work undertaken by an operator. The primary difference between these two closely related terms is that MWL is further mediated by a number of additional factors, including past experience, individual personality traits, and the environment context [12]. For example, a seemingly simple task may not inherently be mentally demanding, but a high level of MWL can be induced if an operator repeatedly performs such a task under additional time constraints. Conversely, a highly complex task may involve a high taskload, but the MWL level experienced may be low if the operator is well-experienced in that particular domain. In summary, while it may be reasonable to use taskload as a proxy for MWL in some contexts, it is critical to recognize that these are fundamentally different, not interchangeable, terms and cannot be equally defined.

Numerous techniques are available to measure MWL in human factors and ergonomics research. Typically, there are three groups of measures: subjective, performance-based, and physiological [13]. Subjective measures rely on self-reported perceptions and are extensively used in practice due to their cost-effectiveness, ease of implementation, and wide acceptance among users [14,15]. However, subjective measures have several drawbacks. For example, some participants may struggle to differentiate between task demands and the mental effort, which can result in underreporting [16]. Furthermore, the subjective measures can be affected by time-delay effects, as information is typically collected post-operation, requiring operators to recall their prior sensations and map them onto a rating scale. Performance-based measures define specific performance metrics to evaluate task effectiveness, such as flight-path deviations in the context of aircraft pilots [17]. Performance degradation serves as an indicator of high MWL, but this method primarily operates reactively and may not meet the requirements of proactively foreseeing potential precursors of increasing MWL to prevent performance deterioration. Advancements in sensing technologies have made it feasible to continuously monitor MWL and detect potential operational risks by measuring human physiological signals using relevant devices [18]. A range of physiological signals, including electrocardiogram (ECG), electroencephalogram (EEG), and electrodermal activity (EDA), have been used in previous studies [19,20]. The main advantage of physiological measures over traditional techniques is their capacity to measure MWL in a continuous manner [21]. This attribute is highly valuable in practical applications wherein instantaneous information about an operator’s MWL is necessary to monitor their mental state in real-time. Additionally, physiological measures provide an objective measurement, which complements the subjectivity inherent in self-reported-based measures. Objectivity is a desirable characteristic for MWL assessment, as it is reasonable to suspect that not all individuals may be able to accurately report their MWL, as noted previously. Furthermore, physiological measures are non-intrusive and require minimal behavioral responses, which are less likely to intervene in primary tasks. Given these advantages, the potential of using real-time physiological signals to measure an operator’s MWL has gained considerable attention from researchers across various fields, including psychology, human factors, and ergonomics.

Several recent reviews have conducted comprehensive investigations into a range of physiological signals for assessing MWL across various domains. For example, ref. [12] investigated the measurement of MWL using multiple physiological signals and provided the evidence base for deploying each measure in practice [22], and also, on the other hand, focused on the multimodal fusion of physiological measures, identifying several potential opportunities for the development of more effective fusion systems for MWL detection. However, the use of various physiological signals poses challenges and may prove impractical to implement in real-flight scenarios due to the potential intrusive effects on pilots and the potential to disturb aircraft instruments [22,23]. Heart rate (HR) and heart rate variability (HRV), primarily derived from ECG, are particularly promising physiological indicators for measuring mental status due to their reliability in detecting changes in autonomous nervous system (ANS) activity, which is strongly linked to elevated MWL [24]. HRV parameters are derived from the oscillations in the intervals between heartbeats, representing the interactions between the ANS and the cardiovascular system [25]. This variability can be either analyzed by change over time or in terms of power spectral density, namely time-domain HRV and frequency-domain HRV [26]. These HRV indicators can detect specific variations in ANS activity when operators are under fatigue, stress, vigilance, or high MWL states. Such alteration, as indicated by HRV indicators, could reflect the brain–heart interaction. The successful application of HRV for MWL measurement has been reported across a range of safety-critical domains, such as pilot [27], air traffic control [28], driving [29], and nuclear plant operation [30]. HRV indicators have also demonstrated correlations with time-on-task effects and the mental resources demanded by specific tasks [31]. Recent systematic reviews of HRV-based driver fatigue and drowsiness detection systems have concluded that HRV indices are promising in detecting these critical mental states [32,33].

Although a large body of research has shown the effectiveness of several HRV indices in the detection of fatigue and drowsiness, there is a lack of agreement on how these measures respond to varying MWL levels, especially within the context of complex pilot operations. Furthermore, most existing studies have focused on drivers, with only a limited number of studies conducted in the domain of aviation piloting [5,34]. Although it might be argued that both driving and piloting share similarities in terms of the attentional resources required and mental demands, they inherently differ in several key aspects. The configuration of instruments inside a cockpit make them more challenging to operate compared to the instrumentation within a car. From an acoustic perspective, pilots are subject to a higher level of attentional demand than normal drivers as they engage in frequent communication with the air traffic management system. Additionally, fatigue, drowsiness, and MWL are essentially different psychological constructs with different underlying mechanisms, making it inappropriate to treat them as interchangeable terms. Another key aspect of pilot MWL measurement is the establishment of MWL prediction models. An accurate MWL prediction is of critical importance due to its role in developing real-time MWL monitor systems, capable of anticipating abnormal mental states of pilots and thus mitigating the risk of human error-related accidents. Traditional statistical-based methods prove inadequate in capturing the intricate and nonlinear relationship between MWL and HRV signals. In contrast, machine-learning-based algorithms have shown promising performance in detecting different levels of MWL based on HRV features. Despite these advancements, there does not yet exist a systematic review covering the application of machine-learning techniques for pilot MWL prediction.

In order to address the aforementioned research gaps, the primary objective of this paper is to provide a comprehensive synthesis of the current literature related to the assessment of pilot MWL using HRV. This review concentrates on the linear HRV metrics in the time and frequency domains due to their simplicity and common usage in existing research, while non-linear metrics are excluded because they are less frequently employed. In particular, this synthesis is intended to achieve the following key aims: (1) systematically explore the various experimental designs employed in prior studies and discuss their potential influence on the resulting HRV responses; (2) provide a comprehensive summary of the responses exhibited by different HRV indices under varying levels of MWL among aircraft pilots; (3) undertake an in-depth review of the recent advancements in machine-learning-based models for predicting pilot MWL, thereby shedding light on the potential for enhancing the performance of MWL assessment through technology; (4) provide valuable insights into the directions for future research endeavors within this domain by synthesizing existing knowledge and identifying potential gaps. Ultimately, this systematic review not only seeks to advance our understanding of pilot MWL measurement but also to pave the way for the development of more efficient automation systems designed to detect fluctuations in pilot MWL.

## 2. Materials and Methods

### 2.1. Literature Search Strategy

The literature search was performed on three databases, namely PubMed, Scopus, and Web of Science Core Collection, to extract the relevant literature. The final database search was performed on 18 May 2023 and the search results were limited to publications from the year 2000 until May 2023, given the rapid advancements in computing and wearable technology. To focus on the specific areas of interest, the following search algorithm was employed for all three chosen databases: “(mental workload OR cognitive workload OR workload OR load) AND (physio* OR ECG OR electrocardiogram OR heart rate OR HR OR HRV OR cardiovascular) AND (flight OR aviation OR aircraft)”. The terms were searched for in the fields of title, abstract, and keywords. The search terms were applied to the fields of title, abstract, and keywords.

### 2.2. Procedure and Eligibility Criteria

**Figure 1:**
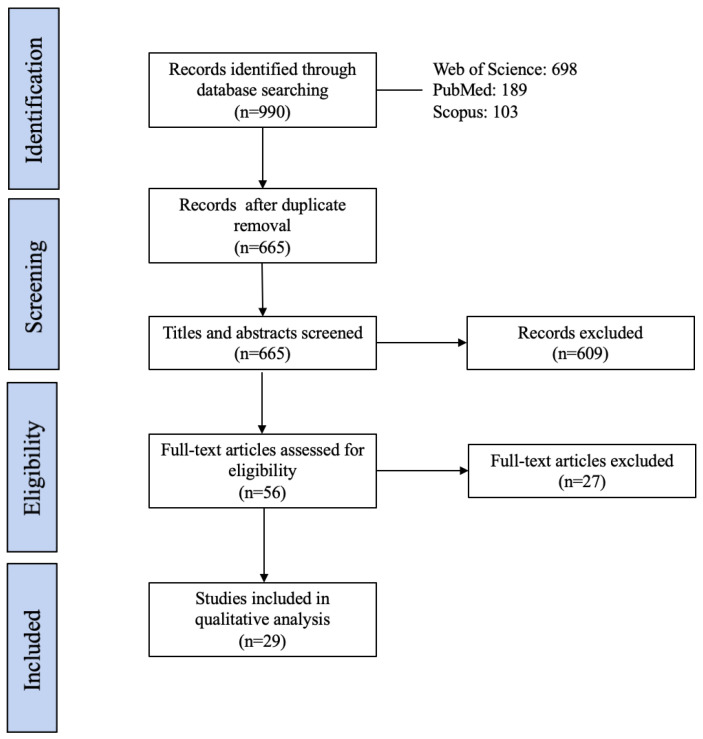
PRISMA flow chart of the literature selection process.

The search and selection process adhered to the guidelines established by the Preferred Reporting Items for Systematic Reviews and Meta-Analyses (PRISMA) framework [35]. A flow diagram adapted from PRISMA, as shown in Figure 1, demonstrates the full search and selection process. The initial search across three selected databases yielded a total of 990 papers. After removing duplicate entries, 665 distinct records remained for further consideration. Subsequently, a rigorous evaluation of the titles and abstracts of these records led to the identification of 56 articles for further in-depth, full-text examination. After carefully reviewing the full texts of these potentially relevant papers, a total of 29 papers were included in this review, and 27 articles were excluded from the full-text assessment based on the defined inclusion and exclusion criteria shown in Table 1. Among the 27 excluded articles, 12 did not focus on flight-related tasks, 7 did not use ECG-based devices to measure heart activity, 3 primarily studied the relationship between HRV indices and performance, 4 were review studies, and 1 article included subjects with health issues. Note that out of the total of 85 references cited in this paper, the remaining references were used to support the introduction and other sections and not for the purpose of the systematic review.

### 2.3. Data Extraction

In our study, we extracted data from a total of 29 relevant research articles. Specifically, the extracted data cover the following aspects: (1) subject characteristics, including information about the number of subjects, their demographic details, and their level of flight experience; (2) flight task settings, which included the type of flight tasks (i.e., simulated or real flight), flight task settings, and the methods used to manipulate MWL; (3) measurement methods, consisting of devices to measure heart activity, and reference MWL measurement approaches; (4) the reported variations in HRV measures in response to elevated MWL; and (5) the machine-learning techniques employed (if applicable) and features used, model selection, and the corresponding model performance. Table 2 presents an overview of the HRV measures included in this review, along with their brief descriptions and investigated frequency in the selected articles. All these measures are standard HRV features that comply with the Task Force guidelines for HRV-related metrics and have been extensively employed in MWL studies and other relevant domains [36].

## 3. Results

Table 3 presents the details of the 29 studies included in this systematic review. The main findings summarized from the literature are organized as follows: First, Section 3.1 examines the diverse experiment designs across the literature. To be specific, we discuss the characteristics of the subjects, flight task settings, and approaches used to manipulate different MWL levels. In Section 3.2, we review the measurements, including the devices used to measure heart activity and the subjective measures used to measure perceived MWL. Then, the findings of the HRV indices used in the pilot MWL assessment are presented in Section 3.3. Finally, Section 3.4 summarizes and discusses the machine-learning techniques used in the included studies and their performance.

### 3.1. Experimental Design

#### 3.1.1. Subjects Characteristics

The sample sizes used included articles ranging from 7 to 35. Approximately one-third of reviewed studies had sample sizes less than or equal to 10. Only two studies used a relatively large study sample with more than 30 participants [37,38]. Age has long been reported as a major contributing factor to heart activity and thus could influence the majority of the HRV results [39]. Among the 29 included studies, there were only three studies that used samples with a wide age range [40,41,42]. No reviewed article compared the difference in HRV responses between different age groups. Another important factor that might influence HRV responses is the experience level of the pilot. Most reviewed studies used professional pilots, and only 3 studies selected participants with no flight experience [38,43,44]. The experience levels of the subjects significantly differ in terms of flight hours, which is a metric to quantify the experience level used by the majority of the studies. In addition, several studies have shown that subjects with different flight experiences can show different physiological responses when performing the same task. Ref. [43] investigated the difference in HR changes between novice and experienced pilots in a simulated environment, and the results showed that the less experienced group had higher HR when compared to pilots with more flight experience. A similar result was also obtained in [45], but it was only significant in the takeoff phase, which has been considered as one of the most information-loaded flight segments. Ref. [46] found the variation of HR was not significant between experienced and less experienced pilots, which is not consistent with previous studies, although a larger sample size is needed to further confirm this finding.

#### 3.1.2. Flight Task Settings

Over two-thirds of the reviewed studies were conducted in a flight simulator, and only nine reviewed articles were performed in a real-flight environment [27,45,47,48,49,50,51,52,53]. Notably, there were two studies conducted in both scenarios and they empirically compared the difference between simulator and real flight [47,52]. Although most studies were performed based on simulation, the flight simulators they used significantly differed in their levels of fidelity, which indicates to what extent the simulation can be comparable to the real world [54]. The type of simulator can be categorized into five groups, ranging from computer screen-based simulators to full motion flight simulators according to previous studies [9]. Only two studies [17,41] used a full motion simulator with six degrees of freedom, which is characterized as the highest level of fidelity. MATB, which was used by four reviewed studies, is a simple, multi-tasking test battery, consisting of four subtasks, namely communication, resource management, tracking, and monitoring. It has been widely used in studying the MWL of non-pilot subjects and multi-tasking [55,56], but may not be sufficiently comparable to real piloting operations.

#### 3.1.3. Mental Workload Manipulation

Significantly different methods have been employed to manipulate MWL levels. Most of the reviewed studies used task load as a close proxy to MWL and manipulated workload by introducing different task difficulties (e.g., high task difficulty is associated with a high MWL level). It is generally assumed that different brain states will be activated by changing the task difficulties, and consequently eliciting different MWL conditions. In the context of flight, the task difficulty levels can be naturally represented by different flight segments. For example, take-off and landing operations are typically considered as more demanding maneuvers than others [57]. The majority of the studies used this approach without explicitly defining different MWL scenarios, where the flight can be divided into several segments, each consisting of several flight maneuvers, and each flight segment can be assigned to a certain MWL level. Additionally, a total of nine studies manipulated MWL conditions by introducing one or more subtasks to increase the mental resources demanded by the tasks.

Different MWL levels can be induced by adjusting the number of subtasks required to complete simultaneously [44], the difficulty of the subtask [43,56,58,59], the occurrence frequency of the subtasks [42,60], or the combination of the subtask with other factors [43,52,61]. The subtasks used in different studies also significantly differ. Several studies opted to use flight-related subtasks. For example, in [52], participants were required to change the responder settings in the cockpit in addition to the primary flight task. Ref. [59] asked subjects to monitor several flight indicators presented on the screen during the cruise phase. Refs. [43,61] employed traditional psychological tasks, such as n-back and mental arithmetic, as additional cognitive tasks. Several studies induced higher MWL levels by generating several events to increase the stimuli, such as engine failure and pump failure [38,47,62,63]. In addition, there were studies that introduced environmental factors, such as low visibility, crosswind, and turbulence, to accelerate the development of high MWL [41,46,61,62,63].

### 3.2. Measurement

#### 3.2.1. Heart Measurement Devices

Different types of devices to measure heart activity have been used in the reviewed studies. Traditional EEG with a number of gel electrodes was used by the majority of the studies. With the advances in wearable and sensing technologies, wearable ECG-based device provides another solution, which was used by six of the reviewed studies [38,41,44,45,61,62].

#### 3.2.2. Reference MWL Measurement

Different measurements were used to obtain the MWL level as the reference. Subjective measure is the most frequently used approach among all included studies. The increased subjective score indicates that an elevated level of MWL experienced by the subject during task execution is elicited successfully. The NASA-Task Load Index (NASA-TLX) [64], which is a well-validated subjective questionnaire, was the most-used subjective measure in the reviewed studies. It is a multidimensional measuring scale that can reflect the overall MWL as well as six subscales, namely mental demand, physical demand, temporal demand, frustration, effort, and performance, and thus it can diagnose the source of elevated MWL [64,65]. Unidimensional scales, such as the Rating Scale of Mental Effort (RSME) and Modified Cooper–Harper Workload Rating Scale (MCH), were also used by several studies due to their simplicity and effectiveness. Seven studies used expert ratings, where experts assessed the participants’ MWL based on their observations and predefined criteria, instead of relying on participants’ self-reports [37,41,46,49,50,66,67]. It was observed that there were several discrepancies between the expert rating and the subjective measures. These discrepancies may be because different groups may have distinct understandings of the MWL, and it could be difficult for some participants to discriminate task difficulty and their MWL, which may result in bias. Notably, [62] used a third-party software that can map the EEG signals to numerical MWL ranging from 0 to 100. This technique provides a solution to continuously obtain the reference MWL level in an extremely short time period. However, the validity of such an approach needs to be tested in the future.

**Table 3:** Overview of reviewed studies.

Reference	Subjects	Flight Task Settings	Measurements	HRV Indicator
[57]	**Size:** 19. **Age:** range 17–27. **Experience:** had prior experience in a simulator, but had not yet started their military flight training.	**Type:** Flight simulator. **Task:** Six flight segments consisting of 37 elements, IFR with simulated meteorological conditions. **Manipulation:** different flight maneuvers.	**Device:** Conventional ECG. **Reference:** RSME.	HR+ MF− HF−
[53]	**Size:** 10. **Age:** mean age 43, range 30–64. **Experience:** mean flight hours 1317, range 158–5400.	**Type:** Real flight. **Task:** Twenty-two flight segments, VFR, IFR, and high-speed IFR (pilots wore goggles that restricted their vision to simulate IFR). **Manipulation:** different flight maneuvers.	**Device:** Conventional ECG. **Reference:** Bespoke measure.	HR+ MF n.s HF n.s
[52]	**Size:** 20. **Age:** mean age 23.3. **Experience:** candidates of the air force.	**Type:** Real flight and fixed-base flight simulator. **Task:** Six segments with increasing levels of difficulty, 19 timed instructions. **Manipulation:** different flight maneuvers and subtask.	**Device:** Conventional ECG. **Reference:** RSME.	HR+ MF− HF−
[43]	**Size:** 12. **Age:** mean age 25. **Experience:** no experience.	**Type:** Computer screen-based simulator. **Task:** Follow a dynamic target with the piloted aircraft, four experimental sessions resulting from the manipulation of two levels within two factors. **Manipulation:** the difficulty of control and subtask.	**Device:** Conventional ECG. **Reference:** NASA-TLX.	LF/HF−
[46]	**Size:** 15. **Age:** range 25–34. **Experience:** Set 1: less experienced (<300 flight hours); Set 2: well experienced (>300 flight hours).	**Type:** Flight simulator. **Task:** Combat flight mission consisting of 13 phases, including beyond visual range interceptions with multiple enemy aircraft and interception of enemy aircraft formation, ILS approach and landing were performed in minimal weather conditions, with no takeoff operation. **Manipulation:** different flight maneuvers.	**Device:** Conventional ECG. **Reference:** Expert rating.	HR+ ΔHR+
[17]	**Size:** 23. **Age:** mean age 31.8. **Experience:** mean flight hours 633.1.	**Type:** Full motion simulator. **Task:** Two segments: (1) take-off and climb (2) ILS approach and landing phase. **Manipulation:** different flight maneuvers.	**Device:** Conventional ECG **Reference:** NASA-TLX.	LF/HF+ SDNN−
[41]	**Size:** 7. **Age:** mean age 48.7, range 35–61. **Experience:** well-experienced.	**Type:** Flight simulator. **Task:** Three segments (take-off phase, in-flight phase, approach, and landing phase) consisting of 18 sessions, each session lasting for 5 min. **Manipulation:** different flight maneuvers and environmental factors.	**Device:** Wearable ECG. **Reference:** NASA-TLX. and expert rating.	HF− LF− LF/HF+ SDNN+ NN−
[48]	**Set 1 (experienced):** **Size:** 4. **Age:** mean age 47.8. **Set 2 (novice):** **Size:** 8. **Age:** mean age 33.1.	**Type:** Real flight. **Task:** Preflight check, take-off, three standard traffic patterns each followed by a touch-and-go landing and takeoff, and final approach and landing. **Manipulation:** different flight maneuvers.	**Device:** Conventional ECG. **Reference:** Unknown.	HR+
[40]	**Size:** 10. **Age:** mean age 44.5, range 28–58. **Experience:** mean flight hours 9025, range 1000–25,000.	**Type:** Flight simulator. **Task:** Four segments (take-off, cruise, ILS approach, and landing) consisting of 24 flight activities, 25–30 min each segment. **Manipulation:** different flight maneuvers.	**Device:** Conventional ECG. **Reference:** NASA-TLX.	HR+ ΔHR+ RMSSD−
[59]	**Size:** 12. **Age:** range 23–25. **Experience:** highly trained but no real-flight experience.	**Type:** Flight simulator. **Task:** Three segments (take-off, cruise, and landing); subjects were required to continuously monitor the flight indicators presented on the head-up display during the cruise phase. **Manipulation:** the difficulty of the subtask.	**Device:** Conventional ECG. **Reference:** NASA-TLX.	HR n.s
[44]	**Size:** 26. **Age:** mean age 20.5. **Experience:** no flight experience.	**Type:** Computer screen-based simulator. **Task:** Simulate multitasking during flight, including the flight target tracking task, the meter monitoring task, the emergency handling task, and the residual capacity task. The residual capacity task is a secondary task, and the other three tasks are primary tasks. **Manipulation:** the number of subtasks.	**Device:** Wearable ECG. **Reference:** NASA-TLX.	HR+ SDNN n.s RMSSD n.s LF/HF n.s
[58]	**Size:** 20. **Age:** mean age 22.7. **Experience:** mean flight hours 141.3.	**Type:** Fixed-base flight simulator. **Task:** From take-off to landing; the established flight paths required various changes in heading, speed, and altitude specifications to vary the course. During the cruise phase, subtasks were introduced. **Manipulation:** the difficulty of the subtasks.	**Device:** Conventional ECG. **Reference:** None.	HR+ SDNN n.s RMSSD n.s pNN50 n.s
[38]	**Size:** 30. **Age:** mean age 34.3. **Experience:** not professional pilots; 4 participants had some flying experience.	**Type:** MATB simulator. **Task:** Four subtasks (the resource management task, the tracking task, the system monitoring task, and the communication task) with two MWL levels. **Manipulation:** the number of events in each task.	**Device:** Wearable ECG. **Reference:** NASA-TLX.	HR+ SDNN n.s VLF n.s LF n.s HF n.s
[68]	**Set 1 (experienced):** **Size:** 4. **Age:** mean age 47.8. **Set 2 (novice):** **Size:** 8. **Age:** mean age 33.1.	**Type:** Computer screen-based simulator. **Task:** Preflight check, take-of, three standard traffic patterns each followed by a touch-and-go landing and takeoff, and final approach and landing. **Manipulation:** different flight maneuvers.	**Device:** Conventional ECG. **Reference:** SOAP.	HR n.s
[66]	**Size:** 14. **Age:** 25–34. **Experience:** mean flight hours 885.	Same settings as [46]	**Device:** Conventional ECG. **Reference:** Expert rating.	
[61]	**Size:** 16. **Age:** 25–34.	**Type:** Fixed-base flight simulator. **Task:** Three phases, including take-off, cruise, and landing, with four MWL conditions. **Manipulation:** visibility and subtasks.	**Device:** Wearable ECG. **Reference:** None.	
[62]	**Size:** 13. **Age:** mean age 36. **Experience:** mean flight hours 605.	**Type:** Computer screen-based simulator. **Task:** Only takeoff phase. **Manipulation:** different events and environmental factors (visibility, weather, wind).	**Device:** Wearable ECG. **Reference:** None.	
[51]	**Size:** 11. **Age:** mean age 21.4. **Experience:** mean flight hours 68.	**Type:** Real flight. **Task:** Three phases (takeoff, downwind, landing) with 2 runs. **Manipulation:** different flight maneuvers.	**Device:** Conventional ECG. **Reference:** NASA-TLX.	
[60]	**Size:** 15. **Age:** range 22–25. **Experience:** no flight experience.	**Type:** MATB simulator. **Task:** Four subtasks (the resource management task, the tracking task, the system monitoring task, and the communication task) with two MWL levels. **Manipulation:** occurrence frequency of subtasks.	**Device:** Conventional ECG. **Reference:** None.	
[49]	**Size:** 10.	**Type:** Real flight **Task:** Twenty-two flight segments with three MWL levels. **Manipulation:** different flight maneuvers.	**Device:** Conventional ECG. **Reference:** Bespoke measure and expert rating.	
[50]	Same as [49]			
[67]	**Size:** 27. **Experience:** mean flight hours 627.	**Type:** Fixed-base flight simulator. **Task:** A number of instrument landing system approaches with a set of subtasks **Manipulation:** temporal demands.	**Device:** Conventional ECG. **Reference:** Expert rating, NASA-TLX, MCH.	NN−
[37]	**Size:** 35. **Experience:** mean flight hours 598.	**Type:** Fixed-base high-fidelity flight simulator. **Task:** ILS approach and additional flying-related subtasks. **Manipulation:** Increasing the load on the subjects by reducing the range at which they commenced the approach.	**Device:** Conventional ECG. **Reference:** Performance.	HR+ NN− SDNN− NN50− pNN50−
[45]	**Set 1 (experienced):** **Size:** 9. **Age:** mean age 33.8. **Experience:** flight hours 487–2883. **Set 2 (novice):** **Size:** 9. **Age:** mean age 23.1. **Experience:** flight hours 220–240.	**Type:** Real flight. **Task:** Preflight check, take-off, three standard traffic patterns each followed by a touch-and-go landing and takeoff, and final approach and landing. **Manipulation:** different flight maneuvers.	**Device:** Wearable ECG. **Reference:** None.	HR+
[27]	**Size:** 17.	**Type:** Real flight. **Task:** One basic airland portion (transporting cargo from one airstrip to another with a high cruise altitude) and one tactical airland part (transporting cargo at low altitudes to an assault landing strip with simulated threats). **Manipulation:** different flight maneuvers.	**Device:** Conventional ECG. **Reference:** MCH.	HR+
[63]	**Size:** 10. **Age:** mean age 37.8. **Experience:** mean flight hours 115.8.	**Type:** Flight simulator **Task:** Eight segments including takeoff, 3 touch-and-go landings, high-speed approach, instrument flight sequence, rerouting, and 3 simulated failures multi-leg cross-country flight. **Manipulation:** different flight maneuvers with flight failure and low visibility conditions.	**Device:** Conventional ECG. **Reference:** Mackworth’ clock test and KSS.	NN− SDNN− LF/HF+
[47]	**Size:** 11. **Age:** mean age 24.8, range 23–28. **Experience:** mean flight hours 156.	**Type:** Real flight and Fixed-based flight simulator. **Task:** Fifteen segments including takeoff, rejected takeoff, engine failure, cruise, instrument approach, and landing. **Manipulation:** different flight maneuvers with engine failure.	**Device:** Conventional ECG. **Reference:** Likert scale.	HR+
[56]	**Size:** 15. **Age:** mean age 21.1, range 18–24. **Experience:** no flight experience.	**Type:** MATB simulator. **Task:** One-dimensional tracking, system monitoring, and resource management. **Manipulation:** the amplitude of the tracking task.	**Device:** Conventional ECG. **Reference:** NASA-TLX.	LF− HF− LF/HF n.s
[42]	**Size:** 7. **Age:** range 19–26. **Experience:** no flight experience.	**Type:** MATB simulator. **Task:** Four subtasks (the resource management task, the tracking task, the system monitoring task, and the communication task) with two MWL levels. **Manipulation:** occurrence frequency of subtasks.	**Device:** Conventional ECG. **Reference:** None.	NN−

For HRV indicator, ‘+’ and ‘−’ denote higher and lower values under elevated mental workload, ‘n.s.’ denotes non-significant result, and fields left empty indicate that they were not investigated in the study. Abbreviations: HRV, Heart Rate Variability; ECG, Electrocardiography; RSME, Rated Scale of Mental Effort; HR, Heart Rate; VLF, Very Low Frequency; LF, Low Frequency; MF, Middle Frequency; HF, High Frequency; VFR, Visual Flight Rules; IFR, Instrument Flight Rules; NASA-TLX, National Aeronautics and Space Agency-Task Load Index; ILS, Instrument Landing System; NN, Normal Normal; SDNN, Standard Deviation of the Normal Normal; RMSSD, Root Mean Square Standard Deviation; MATB, Multi-Attribute Task Battery; SOAP, Sustained Operations Assessment Profile; MWL, Mental Workload; MCH, Modified Cooper–Harper Workload Rating Scale; KSS, Karolinska Sleepiness Scale.

### 3.3. HRV Responses

The majority of the reviewed studies investigated how HR or HRV indices were correlated with increased MWL levels, i.e., the direction of movement in various variables when MWL became higher. In total, 22 studies reported differences in HR or HRV indices in response to varying MWL levels. We commence with an examination of HR in Section 3.3.1, followed by the presentation of HRV results in Section 3.3.2.

#### 3.3.1. Heart Rate

Among all of the measures derived from the ECG, HR is the simplest to obtain and analyze. It is a well-known and long-established metric that has been used to study a variety of human information processing activities in both laboratory and operational environments [69]. Indeed, HR was the most widely investigated index in assessing pilot MWL found during the search of the literature. In total, 15 reviewed studies investigated how HR correlates with elevated MWL levels. Incremental heart rate (ΔHR), defined as the change in heart rate from a baseline or resting state to a specific period during flight operations, was also considered a useful index of pilot MWL. Unlike absolute HR, which provides a measure of the heart’s beats per minute, ΔHR specifically captures the variations in HR that occur in response to increased cognitive demands during different flight phases. It was widely agreed that increased MWL leads to an increase in HR (or decrease in NN interval), which has been confirmed both in simulated studies and in real flight, indicating a globally stronger sympathetic activity. Similar to HR, an increase in ΔHR was observed to be associated with increased MWL experienced by the pilot. However, two studies reported inconsistent findings, where the effect of MWL on HR was not statistically significant [59,68]. With respect to different phases of flight, the highest value in HR was observed during the takeoff and landing phase [40,45,48,53]. Furthermore, with the introduction of more demanding elements, such as rejected takeoff, a significant increase in ΔHR was observed, confirming the sensitivity of HR to the varied mental demands [47]. Ref. [53] observed there was inconsistency between HR and the MWL perceived by pilots using subjective measures. The highest MWL self-rating score appeared in the IFR (Instrument Flight Rules) tracking segment rather than the takeoff and landing phase. Similar results have been obtained by [27], where HR showed subtle variations in the simulated emergency segment, whereas the subjective ratings showed it is the most demanding phase of all segments. However, when the mental demands were extremely high, during the touch and go on the icy runway, the HR and subjective ratings were highly correlated [27]. It was also concluded that HR is the unique HRV metric that could distinguish between rest period and task execution [57]. Ref. [46] also found a significant change in HR between different phases of flight during a simulated flight task. HR was reported to be able to differentiate ANS response variations between different flight segments instead of only between the rest and trial periods [37], which replicated the previous study. Ref. [44] observed that HR can discriminate between different flight segments but only between the highest and lowest task demand scenarios. However, there were no significant differences observed in the mean HR between phases in [40,52,68]. Ref. [38] observed that HR can discriminate between different levels of MWL but not task types during a MATB-based simulation.

#### 3.3.2. Heart Rate Variability

Regarding frequency-domain HRV indices, the LF/HF ratio and HF were the most investigated, with a total of 10 reviewed studies, along with other related research [70,71], considering them to be important physiological indicators of MWL. Additionally, other frequency-based HRV metrics, such as LF, MF, and HF, have been extensively investigated. Notably, one study used VLF analysis as a means to evaluate pilot MWL but obtained non-significant results [38]. These diverse frequency HRV components are associated with different branches of ANS activity. For example, LF is considered a joint reflection of both sympathetic and parasympathetic activities, while HF is primarily associated with parasympathetic activity. The LF/HF ratio is typically considered an indicator of the balance between sympathetic and parasympathetic ANS activities. However, the physiological basis for this interpretation of the LF/HF ratio has been challenged [72]. In the context of time-domain HRV, SDNN was the most investigated measure within the reviewed studies as its simplicity in calculation compared to other metrics, directly derived from the NN interval. Ref. [37] tested several time-domain indices and they observed that NN was the only indicator that could differentiate the high-performance group from the sub-standard-performance group. However, it is noteworthy that, overall, time-domain HRV received relatively less attention in studies related to pilot MWL when compared to frequency-domain HRV.

Typically, increased MWL levels induce lower HRV values [73]. However, contradictory findings exist across the literature, especially for the LF/HF ratio (e.g., the direction of change in HRV with elevated MWL was not consistent). For example, a lower LF/HF ratio at the highest level of MWL was only observed in [43], while all other studies reported higher ratios, and two studies did not find significant results [44,56]. Ref. [56] observed significant inter-individual differences in terms of the LF/HF ratios. With increasing mental demands, parasympathetic activity tends to decrease, resulting in concurrent reductions in both LF and HF components. Consequently, the direction of change in the LF/HF ratio becomes less distinct.

Several studies examined HR and HRV parameters simultaneously, and the results consistently demonstrated that HR exhibits greater sensitivity in response to varying mental demands in comparison to HRV. Ref. [53] observed that the changes in HRV were not significant during different flight segments, while HR demonstrated a strong correlation with varying mental demands. Similarly, ref. [58] observed an immediate increase in HR as MWL increases, whereas HRV failed to show significant differences across various MWL conditions. The discriminative power of HR in detecting MWL changes was also confirmed by [38], where HR proved effective while HRV did not. During actual flight scenarios, ref. [51] conducted a comparative analysis of ECG changes across two flight runs. Their findings indicated that HR was the sole indicator sensitive to the “run effect”, with the first run displaying higher HR values compared to the subsequent run. In comparing simulated and real-flight scenarios, HR exhibited significant variations between simulations and real flights, whereas HRV did not exhibit such distinctions. However, regarding the momentary changes, both HR and HRV were identified as responsive indicators to short-term fluctuations in MWL, with the HF band of HRV displaying greater sensitivity [57]. Additionally, ref. [63] revealed a temporal aspect within HRV parameters, with elevated LF/HF ratios persisting for over two hours post-flight, and decreased NN intervals and SD1 values enduring for up to five hours after the flight.

### 3.4. Pilot MWL Detection Using Machine Learning

In total, eight studies developed HRV-based MWL systems to discriminate between different pilot MWL situations, using a range of machine-learning techniques. Table 4 provides a comprehensive summary of the details of these studies. It should be noted that the direct comparison of algorithm performance across these studies is not feasible due to variations in experimental designs. As discussed in the preceding sections, factors such as the utilization of different subjects, variations in flight task configurations, and the specific sensors employed can significantly impact model performance. To ensure a comprehensive 406 overview, we present the review from the following three aspects: problem definition (Section 3.4.1), feature selection (Section 3.4.2), and model performance (Section 3.4.3).

#### 3.4.1. Problem Definition

Most of the reviewed detection systems were defined as a classification problem, where each data sample, comprising the physiological signals measured within a defined time window, was labeled with a numerical representation corresponding to the MWL level (e.g., low MWL designated as 0 and high MWL as 1). However, it is noteworthy that two studies departed from this classification paradigm and instead treated MWL assessment as a regression problem, employing a quantified numerical range to represent MWL levels. Overall, in the case of binary classification, these systems have demonstrated promising classification performance, with the highest reported accuracy exceeding 90% [60]. Notably, in the context of multi-class classification, ref. [61] built multi-class MWL classifiers using a support vector machine (SVM), but the results were not satisfactory, with the highest accuracy falling below 50% for three-class classification and even lower for four-class classification. Similarly, ref. [49] reported the inherent difficulty of classifying MWL into multiple classes compared to binary classification. Nevertheless, they argued that the primary objective of the MWL detection system lies in the accurate identification of high MWL levels, and thus it is not practically meaningful to detect moderate MWL conditions. This statement should be questioned because multi-class classification has the potential to anticipate a rising trend in pilot MWL. This capability allows for timely and tailored adaptative aiding to prevent MWL from going beyond the “high MWL” class. In essence, multi-class classification can facilitate a more proactive approach to MWL management.

#### 3.4.2. Feature Selection

Using a single-signal approach to construct machine-learning models was typically regarded as less reliable, primarily due to the likelihood of these encoded features failing to capture sophisticated relationships. However, ref. [66] achieved acceptable results using only one HRV measure. In contrast, ref. [60] highlighted the importance of leveraging multiple HRV features to develop a MWL classifier with high accuracy. In studies adopting a poly-signal approach, EEG and eye-related measures were the most used, together with ECG. Using a poly-signal approach can consistently yield robust model performance, as demonstrated by [51], where the introduction of eye-related features substantially enhanced classifier accuracy. Ref. [61] used principal component analysis (PCA) to identify the most significant features within an extensive set, including 623 features derived from ECG and EEG, and results showed PCA can significantly improve the performance while reducing the computational demands. Alternatively, ref. [50] proposed a feature combination strategy to linearly fuse HR and eye-related signals into one single measure. This feature combination has demonstrated the potential to significantly improve learning performance, from approximately 50% to 80% in terms of classification accuracy. However, this reconstruction may sacrifice the interpretability of the original features in further feature analysis [74].

#### 3.4.3. Model Performance

It is important to note that there does not exist a universal model capable of consistently outperforming others across all scenarios. The performance of these models is largely dependent on a range of factors, including data quality, the nature of input features, and the strategies employed for training and validation. The primary objective of this section is to summarize the diverse models employed in previous research and provide insights into future research directions concerning model selection. Regarding traditional machine-learning techniques, SVM has emerged as a widely utilized model. It has been reported that SVM consistently achieves superior model performance when compared to other machine-learning methods [60,61]. However, these promising outcomes have predominantly been observed in binary classification scenarios, specifically in the discrimination of low and high MWL states. Regarding deep-learning models, multilayer perceptron (MLP), characterized by three layers of neurons, has also demonstrated substantial promise in MWL classification tasks [42,49,50]. The inherent strength of MLP lies in its capacity to adeptly approximate complex and multidimensional non-linear functions, attributable to its parallel architecture [75]. However, neural networks, including MLP, are often characterized as ’black-box’ models, which are less interpretable when compared to traditional statistical-based, machine-learning models. One included research developed a personalized model to classify different MWL levels, and the results suggested that the model trained at an individual level can consistently realize better performance than the collective model trained at the whole population level [60].

## 4. Discussion

### 4.1. Primary Findings

Table 5 summarizes the key insights derived from the reviewed literature, categorized according to each respective section.

#### 4.1.1. Experiment Design

Significant variations in study design were observed across all the articles under review. These diverse experimental designs introduce complexities when attempting to quantitatively interpret and compare results across different studies. Notably, concerning the study populations, the sample sizes in the reviewed articles appeared relatively small compared to other similar research, such as studies on driver fatigue. For instance, a recent review by [32] reported sample sizes ranging from 2 to 86 in studies related to driver fatigue detection using HRV indices. This discrepancy in sample size may be attributed to the inherent challenges of recruiting participants from the professional or well-experienced pilot population. In contrast, the driver’s operating environment is generally less complex than that of a pilot, enabling more convenient data collection on a larger scale. Nevertheless, the limited sample size reported in the reviewed articles may compromise the reliability of the results. To enhance the robustness of the experimental findings, it is necessary to conduct more extensive studies involving larger and more diverse participant cohorts. Age potentially constitutes a significant confounding factor in the examination of the causal relationship between elevated MWL and various time and frequency domain HRV indices. Remarkably, none of the studies included in our review explicitly addressed the influence of age on HRV measures. Furthermore, empirical evidence indicates that flight experience is correlated with HRV responses, as more experienced operators tend to exhibit lower MWL levels when performing the same tasks. Nevertheless, it is worth noting that the age of the subjects could serve as a confounding variable, given that more experienced pilots typically have a higher age profile than their less experienced counterparts. Thus, the reported relationship between experience level and HRV responses should be interpreted with caution, and future research should undertake a more comprehensive validation of previous results by considering potential confounders such as age.

Regarding the ECG devices used, unlike conventional ECG, which typically requires professional assistance, the wearable ECG can offer additional usability and convenience for operators as they can be easily operated by the participants themselves [45,76]. It can also reduce the underlying gap between laboratory settings and real-world applications. In addition to ECG, photoplethysmogram (PPG)-based solutions can also be used to measure HR-related signals. PPG-based measures are relatively simple and convenient as they can be integrated into wristbands [77]. However, the signal quality is highly likely to be contaminated by motion artifacts, which can result in incorrect beat intervals and reduce the detection performance [65]. Thus, studies that used PPG-based devices were excluded from this review.

#### 4.1.2. Use of HRV in a Real-World Scenario

The majority of the included studies employed flight simulators rather than real flights. Using a simulator is more convenient and effective for organizing large-scale experiments to obtain a large amount of data. The simulator can provide a more controllable environment as different levels of task difficulty can be easily and systematically set in the simulator, and the real-world task can be affected by uncertain factors and confounds [52]. In contrast, real-flight tasks are inherently influenced by a multitude of uncertain environmental factors and confounding variables. Given these advantages of simulators, however, the findings from these studies may face challenges when applied in real-world setups. This is because a simulated flight task may inherently involve lower mental demands compared to real-flight operations due to the absence of real-world consequences, such as the risk of collision and injury, even when emergency scenarios are intentionally introduced [37]. Furthermore, it is essential to recognize that the physical conditions within a real cockpit add additional complexities. Factors like extreme cockpit temperatures and high gravitational forces can exert significant effects on physiological responses during real flights, as highlighted by [67]. Thus, the results obtained from simulated studies may lead to potentially misleading conclusions and may offer insufficient insights into the physiological responses associated with elevated MWL. Therefore, it is essential to further investigate the actual applicability and validity of these physiological measures in real-world flight scenarios.

#### 4.1.3. Considerations of Simulator Fidelity

The fidelity of a flight simulator is also a crucial consideration, as varying levels of fidelity can influence physiological responses, MWL, and flight performance. To obtain a comprehensive understanding of how HRV responds to heightened MWL, it is important to investigate the impact of simulator fidelity on MWL. Directly quantifying fidelity from the simulator poses significant challenges. A practical approach involves comparing real-world flight experiences with similar task settings conducted within a simulator using both subjective assessments and objective metrics to infer simulator fidelity. Given it is challenging for researchers to collect data from real flights as it is essential to ensure that data collection does not have a negative impact on safety [78], a favorable approach is to use high-fidelity flight simulators that can simultaneously ensure safety and maintain data quality. Furthermore, a significant challenge in cross-study comparisons lies in the heterogeneity of MWL manipulation methods. Most reviewed studies employed different task difficulties to induce distinct MWL scenarios. However, it is essential to recognize that increased task difficulties may not always lead to increased MWL correspondingly. For instance, in situations where participants experience low SA, they may struggle to gather sufficient information, potentially failing to engage in high-level mental processing. In such cases, even with high task demands, participants may still contend with lower MWL levels [67].

#### 4.1.4. Measurements

Regarding the heart monitor devices, traditional ECG was the most used, while several studies opted to use wearable ECG devices such as ECG chest straps or ECG shirts. Traditional ECG devices with multiple leads and high-quality sensors might be more accurate in capturing electrical signals regarding cardiac electrical activity. Additionally, these devices have long established their reliability and validity in clinical settings for diagnosing a range of cardiac conditions compared to wearable ECG. Nevertheless, traditional ECG-based devices normally rely on wet gel electrodes, which operate by incorporating a conductive gel layer between the electrode and the skin. This method can result in a messy application and lacks practicality for routine use in occupational settings. In contrast, wearable ECG devices offer a desirable feature: the potential ability to measure HRV in real-world aviation scenarios, providing crucial insights into pilots’ physiological responses during actual flight operations. Moreover, these wearable devices are characterized by their compactness, lightweight design, and comfort during extended flights. These features ensure that pilots can wear them without distraction, discomfort, or interference with their duties. Thus, future research should undertake more validations and explorations of the applicability of wearable ECG devices within real-world flight settings, particularly in assessing elevated MWL and its interaction with diverse environmental factors affecting the ANS.

Regarding the measurement of reference MWL, multidimensional questionnaires, such as NASA-TLX, were the most widely used due to their established validity, reliability, sensitivity, and diagnostic capacity. However, in practical flight scenarios, the adoption of unidimensional scales may prove advantageous, offering instantaneous MWL information. Ref. [38], for example, employed a single-scale version of NASA-TLX, focusing on the “mental demand” dimension. They pointed out that given the nature of the piloting tasks is primarily mental rather than physical, it is sufficient to capture a pilot’s workload without using the full six-dimensional version. Nevertheless, the sensitivity of the unidimensional measures is suspected by some studies as they may fail to capture the complex cognitive information processing of humans and may lack the capacity to diagnose demands on different cognitive modalities. It is important to acknowledge that subjective MWL measurements inherently lack objectivity, potentially resulting in dissociation from objective measures. Furthermore, it is challenging to balance between the intrusiveness of real-time implementation and the retrospective bias introduced by post-task evaluation, as highlighted by [10]. To address the limitations of subjective measures, alternative, more objective approaches have been introduced, such as expert ratings, where trained individuals or experts assess a pilot’s workload. However, the presence of inter-rater variability among different experts evaluating the same flight or task can compromise the reliability and consistency of expert ratings. Therefore, future research endeavors should strongly consider adopting a multimodal approach in assessing MWL to provide a more comprehensive perspective.

#### 4.1.5. Physiological Responses

Regarding the physiological measures, HR was the most commonly used in these studies. This predominant use of HR can be attributed to its simplicity in measurement and interpretation. In contrast, HRV indices typically either involve analyzing the variations in the time intervals between successive heartbeats or assessing the distribution of HRV across specific frequency bands, which can be more complex and may require specialized equipment and expertise. The majority of the studies found that an increase in HR corresponds to higher levels of MWL, a pattern that aligns with previous research. Nonetheless, it is noteworthy that several studies have observed that the direction of HR change is not always predictable, and it appears to depend on the particular task at hand. This variability in HR response is consistent with findings in traditional psychology and cognition research. For example, HR may decrease during certain tasks, like visual illusionor mental rotation, while it may increase during tasks involving multitasking or additional memory load [79]. HR is known to be influenced by factors such as muscular activity and psychological stress. This can account for the inconsistent HR results observed in two specific studies, where the experiments primarily involved monitoring tasks with a mouse rather than simulating actual piloting. This setup likely resulted in reduced physical exertion and possibly lower levels of anxiety compared to more immersive piloting scenarios, which typically incorporate comprehensive physical and cognitive challenges. The lack of these stimuli in the reviewed studies may explain the deviation in HR results from those seen in studies that involve actual aircraft control [5,59,68].

Regarding the different phases of flight, the highest HR values were observed during the take-off and landing stages, indicating elevated information processing demands during these critical phases and thus elevated MWL. However, it is noteworthy that some studies found that these observations did not consistently align with the self-reported MWL scores provided by the pilots. In other words, the phases of flight that are perceived as the most mentally demanding do not consistently correspond to the phases where the highest HR values are recorded. This discrepancy may arise from scenarios that are not encountered frequently by pilots, while take-off and landing represent more routine flight operations. This suggests that HR may be more sensitive to the actual mental demands placed on the pilot, whereas subjective responses may reflect perceived mental demands. Furthermore, several studies have explored using HR to distinguish between different phases of flight. However, the findings in this regard have been somewhat inconclusive. Ref. [40] suggested that this inconsistency could be due to the heterogeneous physiological responses exhibited by individuals during specific flight tasks. Additionally, the inherent nature of the task itself may contribute to this inconsistency. Highly demanding flight tasks may elicit a greater degree of variation in ANS activity, leading to more obvious fluctuations in HR values.

It is important to note that while HR can provide valuable information about physiological responses to mental workload, HRV indices can offer more detailed insights into ANS activity and mental states. It was found that frequency-domain HRV has gained greater attention when compared to time-domain HRV. This relatively less investigation may be attributed to the inherent limitations of time-domain analysis. This approach, which is based on simple statistics, may be considered insufficient in providing a comprehensive understanding of the temporal structure and periodicity within the data. In contrast, using spectral analysis has been advocated by researchers as it can offer a more sophisticated approach. It decomposes HRV into components, representing the signal series as a summation of sinusoidal components of different amplitudes, frequencies, and phase values [26,80]. This spectral analysis approach provides a more insightful examination of HRV patterns, potentially explaining its greater utilization in the study of pilot MWL.

Although HRV has been considered an important indicator of MWL, several studies have reported contradictory results with bidirectional changes and non-significant results. This inconsistency can be attributed to the heterogeneity of experimental settings. For example, small sample sizes may not adequately represent the broader population, introducing potential biases that limit generalizability. Additionally, HRV has also been reported to increase during monotonous or repetitive tasks [81]. Tasks involving speech present another dimension of complexity, as they can induce changes in blood pressure, which, in turn, influence the MF component of HRV. Neglecting the individual differences in physiological responses may also contribute to these inconsistent results. Furthermore, HRV, particularly its spectral components, can be influenced by respiratory activity, representing a confounding factor that can contaminate HRV measurements. For example, during continuous execution, participants typically engage in deeper and slower breathing, which enhances vagal tone and results in an increase in the HF band of HRV. This is because the HF band is primarily associated with parasympathetic nervous system activity, which is modulated by respiratory patterns [16,82]. Notably, ref. [52] observed substantial HRV fluctuations over time, attributing these unexpected variations to respiratory activity. They demonstrated that as respiratory frequency decreases and amplitude increases, there is a pronounced rise in both the MF and HF bands. Addressing this issue can be approached in several ways. One straightforward method involves excluding HRV values when respiratory frequency falls below or exceeds certain thresholds [52]. However, determining precise and universally applicable threshold remains a challenge. Another approach is to synchronize the respiration rate of participants. Nevertheless, enforcing constant breathing is often impractical in real-world scenarios, such as piloting or driving. In future research endeavors, more robust techniques are desirable, even if the HRV changes were influenced by respiratory activity, as long as they exhibit temporal correlations.

When comparing the sensitivity of HR and HRV during flight scenarios, HR appears to exhibit greater sensitivity to variations in mental demands compared to HRV in both simulated and actual flight situations. This result is in alignment with a previous review on HRV and MWL, concluding that spectral HRV displays limited sensitivity to increased task complexity [21]. This reduced sensitivity of HRV might be attributed to its intricate relationship with respiratory activity, as we discussed earlier. In a comprehensive study examining ten measures, both HR and HRV demonstrated promising results in distinguishing between various levels of MWL, even when task demand variations were subtle and performance scores remained stable [83]. Nevertheless, it is crucial to acknowledge that no single HRV variable consistently serves as a reliable MWL indicator. Several studies have found that HR is a more favorable measure compared to HRV, primarily due to its ease of acquisition, heightened sensitivity to MWL, and a more established validation of the direction of change associated with increased MWL. However, HRV, particularly when analyzed in the frequency domain, can capture unique information about MWL. Furthermore, it should be noted that no measure is reliable at an average level as there is a strong individual difference between MWL and those measures. Therefore, a single HR or HRV measure cannot comprehensively capture the intricate cognitive processing that occurs during flight, emphasizing the need for a combination of HR and HRV in future research. Additionally, investigating these measures at the individual level is of great significance.

#### 4.1.6. HRV-Based MWL Detection System

Multi-classification of MWL is inherently more challenging than binary classification due to the increased complexity introduced by the presence of multiple MWL levels. In binary classification, the task revolves around distinguishing between two clearly defined states, typically low and high MWL. In contrast, multi-class classification necessitates the discernment of MWL across multiple levels, where the distinctions between these MWL levels may be subtler, making it more challenging to establish distinct decision boundaries. Moreover, with an expanded number of classes, the potential for class imbalances and overlapping feature distributions increases, further complicating the classification task. Nevertheless, multi-class classification is valuable as it can provide a more nuanced understanding of MWL variations and enable proactive mitigations to operators under high MWL effectively. It is worth noting that all the models were developed with supervised learning techniques. However, traditional supervised learning requires labeling all the collected data, which is cumbersome and not cost-effective. In light of this, unsupervised or semi-supervised learning techniques with anomaly detection techniques could be explored in future research. One possible strategy for model training is first training the model on data representative of normal MWL or the entire dataset, with a predominant proportion being normal state. Then, these models can be used to identify anomalies within the data, specifically targeting instances representing high MWL scenarios. This type of approach alleviates the labeling burden and facilitates the efficient detection of abnormal MWL states.

In machine-learning algorithm implementation, feature selection is a critical consideration, and different feature selection methods have been used across studies. Regarding the single-signal approach, using multiple HRV indices as input features has the potential to capture more complex relationships compared to using a single HRV. Nevertheless, there are several inherent challenges. First, a strong correlation exists between traditional time-domain and frequency-domain HRV measures, potentially leading to reduced interpretability and generalization performance. Furthermore, HR and HRV can only reflect holistic cardiac rhythm information but cannot capture nuances in ECG wave shape. Non-linear metrics such as total power, sample entropy, and maximum Lyapunov exponent were considered to provide complementary insights, as these capture different aspects of heart rate variability that are not reflected in traditional HR-based features. Employing multiple physiological signals also introduces potential risks, including data sparsity in high-dimensional space, which can hinder model performance and have the risk of overfitting when using an excessive number of features. Dimensionality reduction techniques, such as PCA, are powerful tools to mitigate these issues by effectively reducing the dimensionality of the feature space and preserving essential information while eliminating redundant or noisy features.

The prevailing models employed in prior studies have primarily focused on traditional machine-learning algorithms and fundamental deep-learning architectures. Further exploration could focus on the use of advanced deep-learning tools, such as attention-based mechanisms, and the design of hybrid models with more complex architecture designed specifically for addressing the challenges inherent in MWL detection tasks. Whether these advanced techniques outperform traditional machine-learning models in the context of pilot MWL detection remains an open research question. It is noteworthy that variability in physiological responses to diverse mental states among individuals is well-documented and can be attributed to multiple factors, including personality traits, emotional disposition, prior experiences, sleep quality, etc., [84]. For example, different individuals may employ distinct strategies when confronted with identical scenarios. Differences in skills and past experiences among operators facing the same situations could also potentially lead to varying physiological reactions; what one person might perceive as highly demanding, another might view as a manageable challenge [85]. In order to address the inter-individual differences, several studies have advocated personalized models, which are specifically trained using data from a person. Ref. [86] utilized personalized baseline data to construct a tailored feature set, considering the intrinsic personal difference in HRV measures. Furthermore, one study categorized participants into two distinct age groups and the results implied that a model tailored for a specific age group may not be adequately applicable to another age group. Consequently, the development of personalized MWL detection systems is of great importance, especially in the context of adaptive cockpit design. Such systems have the potential to autonomously monitor pilot MWL fluctuations and provide timely adaptive assistance, such as during periods of extremely low or high workload. However, the practicality of individual training models for each pilot is challenging. Collecting a substantial amount of data for each pilot is not only time-intensive but is also not cost-effective. An alternative approach to enhance the transferability of MWL detection systems across individuals is through the application of transfer learning techniques. With this approach, once a foundational model is established, minimal recalibration is required to adapt it to individual differences. In light of these considerations, future research should delve into comprehensively understanding how individual pilots uniquely respond to varying mental demands. Furthermore, the exploration of transfer learning techniques holds promise in the pursuit of realizing personalized pilot MWL detection systems that are both effective and operationally viable.

### 4.2. Future Considerations

It should be noted that MWL is a multi-dimensional construct in nature and thus it is complex to measure and analyze pilot MWL. This systematic review does not allow us to definitively establish a solid association between HRV and pilot MWL. Nevertheless, HRV exhibits the potential to serve as a promising physiological indicator for assessing pilot MWL in real cockpit settings. To comprehensively understand the relationship between these measures and pilot MWL, further investigations are needed to explore the impact of various factors, including experimental design, measurement methods, and inter-individual differences, on physiological responses.

In practice, it is challenging and vague to establish certain thresholds to determine whether a pilot is experiencing high MWL. Thus, the development of machine-learning models to implicitly predict or classify pilot MWL based on HRV features is a feasible solution. However, as highlighted in previous sections, relying solely on HRV measures may inadequately capture the complex mental processes during flight. It would be more holistic if multiple physiological signals could be recorded simultaneously in a nonintrusive and effective manner. Even with multiple measures, capturing the intricate relationship between pilot MWL and physiological signals can remain a huge challenge. For example, MWL may also be influenced by other psychological constructs, such as anxiety and SA. While these constructs were not the central focus of this review, future studies could explore their interconnectedness with MWL and assess their potential as supplementary measures of MWL.

It is essential to monitor HRV signals in ecologically valid environments before the MWL can be effectively measured using HRV in real flight. Recent technological advancements in virtual reality (VR) have provided such an opportunity for measuring pilot MWL in an ecologically valid scenario. VR enables highly immersive flight simulations closely mimicking real-world scenarios, and it is more cost-effective than traditional flight simulators. This area of investigation holds particular promise, as VR-based flight simulators are still in the early stages of design and implementation. Consequently, questions regarding their effectiveness and assessment of the degree of fidelity required in VR simulations to accurately replicate real-flight conditions necessitate further investigations.

## 5. Conclusions and Future Research Directions

### 5.1. Conclusions

In conclusion, the reviewed studies proved that HRV indices hold the potential to serve as a valuable indicator for the measurement of pilot MWL. However, inconsistent relationships between HRV measures and varying levels of MWL were observed. This variability could be attributed to discrepancies in the underlying study designs and measurement methods employed across different studies. Therefore, future studies are encouraged to develop consistent experiment design protocols and provide more transparent experiment configurations. Furthermore, significant advancements are still needed before HRV-based MWL assessment can be effectively applied in real-flight scenarios, and it is crucial to validate current findings from simulator and controlled flight studies through real-life flight studies for greater generalizability. Additionally, the pursuit of more accurate MWL detection systems may involve unsupervised learning techniques, alternative personalization strategies, and the integration of diverse signals from multiple sources. Future research could also examine the potential of VR-based flight simulators in pilot MWL studies.

### 5.2. Future Research Directions

Based on the results of this review, we suggest the following directions for future research:

1. **Standardization of HRV Measurement Protocols:** Future research should aim to establish standardized HRV measurement protocols to ensure consistency and comparability across studies. This includes standardizing the devices used, the specific HRV metrics measured, and the experimental conditions under which data are collected.
2. **Integration of Multimodal Data:** Combining HRV data with other physiological and behavioral measures (e.g., EEG, eye tracking, and subjective assessments) could provide a more holistic understanding of MWL. Future studies should explore multimodal data integration to enhance the accuracy and reliability of MWL assessment models.
3. **Longitudinal Studies:** Conducting longitudinal studies to examine the temporal dynamics of HRV and MWL over extended periods is crucial. This will help in understanding how MWL fluctuates over time and in different flight conditions.
4. **Real-World Applications:** While many studies have used flight simulators, there is a need for more research in real-world flight conditions. Future studies should validate the findings from simulators in actual flight scenarios to ensure the practical applicability of HRV-based, MWL assessment tools.
5. **Advanced Machine Learning Techniques:** Exploring advanced machine-learning techniques, such as deep-learning and ensemble methods, could further improve MWL detection accuracy. Additionally, developing personalized models that account for individual differences in physiological responses to MWL is another promising direction.

By addressing these directions, future research can build upon the findings of this review and contribute to the development of robust, reliable, and practical tools for assessing pilot MWL in aviation.

## Figures and Tables

**Table 1:** Inclusion and Exclusion Criteria.

Inclusion Criteria	Exclusion Criteria
(1) Written in English.	(1) Review papers or meta-analysis.
(2) Peer-reviewed journals.	(2) Targeted physical workload.
(3) Examined HR or at least one HRV for MWL.	(3) Conducted on subjects with health conditions or diseases affecting HRV.
(4) Included a flight-related task.	(4) Did not use ECG-based device.

**Table 2:** Summary of the HRV indices examined in the review.

Domain	Index	Unit	Description	Frequency
Time	HR	1/min	The number of beats over a given time period	15
	NN	ms	Normal to normal interval. Also known as the RR interval or the interbeat interval (IBI)	5
	SDNN	ms	Standard deviation of normal-to-normal intervals	7
	RMSSD	ms	The square root of the mean squared differences of successive NN intervals	3
	NN50	count	The number of pairs of successive NN intervals that differ by more than 50 ms	1
	pNN50	%	The percentage of NN50 divided by the total number of NN intervals	1
Frequency	VLF	ms^2^	The power of the very low frequency band (less than 0.04 Hz)	1
	LF	ms^2^	The power of the low frequency band (0.04 to 0.15 Hz)	3
	HF	ms^2^	The power of the high frequency band (0.15 to 0.4 Hz)	6
	LF/HF	ratio	The ratio of LF to HF power	6

Count refers to the number occurrences and ratio refers to dimensionless. Abbreviations: HRV, Heart Rate Variability; HR, Heart Rate; NN, Normal to Normal; SDNN, Standard Deviation of the Normal to Normal; RMSSD, Root Mean Square of Successive Differences; VLF, Very Low Frequency; LF, Low Frequency; HF, High Frequency.

**Table 4:** Summary of studies using machine-learning techniques with details.

Reference	Problem	Data/Measures/Features	Model Selection	Best Performance
[66]	Regression	**Ground truth:** Observer rating. **Measure(s):** ECG. **Feature:** NN	**Model(s):** MLP and multiple regression model. **Train and validation:** 8 participants for train, 6 participants for validation	**MAE:** 9.9 (MLP)
[61]	Binary, multi-class classification	**Ground truth:** Low and high MWL induced by different events and environmental factors. **Measure(s):** ECG, EEG. **Feature:** Top 3 HRV measures and top 20 EEG measures obtained by PCA	**Model(s):** LDA, SVM, KNN. **Train and validation:** personalized model	**Accuracy:** 2-class: 75% (SVM) 3-class: 48.21% (KNN) 4-class: 37.2% (LDA)
[62]	Regression	**Ground truth:** EEG-based MWL scoring system (ranging from 0–100). **Measure(s):** ECG, EEG, eye tracker. **Feature:** Top 10 features obtained by Pearson correlation analysis	**Model(s):** Ridge regression, SVM, MLP, CNN, Bi-LSTM, Stacked-LSTM	**MAE:** 5.28 (Stacked-LSTM), **MSE:** 44.09 (Stacked-LSTM), **RMSE:** 6.64 (Stacked-LSTM)
[51]	Trinary classification	**Ground truth:** Low, medium, and high MWL induced by different flight phases. **Measure(s):** ECG, eye tracker. **Feature:** HR, SDNN, fixation duration, saccadic rate, visual entropy	**Model(s):** LDA with all features, LDA with HR, LDA with saccadic rate. **Train and validation:** trained on the first run and tested on the second one using a leave one out cross-validation	**Accuracy:** 75% (LDA with saccadic and LDA with all features)
[60]	Binary classification	**Ground truth:** Low and high MWL induced by the occurrence frequency of subtasks. **Measure(s):** ECG. **Feature:** NN, Total Power, QRS wave power, Sample Entropy	**Model(s):** SVM with linear kernel, SVM with RBF kernel, RF, Adaboost. **Train and validation:** K-fold cross validation; personalized model	**Precision and recall:** 90.88% and 91.86% (SVM with all features trained on individual)
[50]	Binary classification	**Ground truth:** Low and high MWL derived from subjective measure. **Measure(s):** ECG, EEG, eye-related measure, respiration. **Feature:** Features obtained from the combination and calibration scheme with three moving averages	**Model(s):** MLP. **Train and validation:** Leave-one-in strategy	**Accuracy:** 80% (MLP with feature combination and calibration)
[49]	Binary classification	**Ground truth:** Low and high MWL derived from subjective measure. **Measure(s):** ECG, EEG, eye-related measure, respiration. **Feature:** Features obtained from statistical stepwise screening and signal-to-noise ratio saliency	**Model(s):** MLP, quadratic discriminant model, linear discriminant model. **train and validation:** personalized model	**Accuracy:** 82% (MLP)
[42]	Trinary classification	**Ground truth:** Low and high MWL induced by the occurrence frequency of subtasks, plus the baseline scenario. **Measure(s):** ECG, EEG, EOG, respiration. **Feature:** 43 features consisting of 30 EEG channels and 10 EOG channels with five bands each, plus the interbeat, interblink, and respiration intervals.	**Model(s):** MLP. **Train and validation:** 10s window size with 50% overlap; 75% train, 25% test.	**Accuracy:** 85.0% for baseline 82.0% for low MWL 86.0% for high MWL

Abbreviations: ECG, Electrocardiography; NN, Normal Normal; MLP, Multilayer Perceptron; MAE, Mean Absolute Error; MSE, Mean Square Error; RMSE, Root Mean Square Error; MWL, MentalWorkload; EEG, Electroencephalography; HRV, Heart Rate Variability; PCA, Principal Component Analysis; LDA, Linear Discriminant Analysis; SVM, Support Vector Machine; KNN; K-Nearest Neighbors; CNN, Convolutional Neural Network; LSTM, Long Short-Term Memory; SDNN, Standard Deviation of Normal Normal; RBF, Radial Basis Function; RF, Random Forest; EOG, Electrooculographic.

**Table 5:** Summary of Primary Findings.

Section	Findings
Experiment design	Significant variations in study design complicate quantitative interpretation and comparison across studies. Small sample sizes in reviewed articles may compromise reliability. Flight experience correlated with lower MWL levels but was potentially confounded by age.
Use of HRV in a real-world scenario	Majority of studies used flight simulators, which provide controlled environments but may not fully replicate real-world mental demands. Findings from simulator studies may not generalize well to real-world flight scenarios due to the absence of real-world consequences and physical conditions.
Considerations of simulator fidelity	Simulator fidelity impacts physiological responses and MWL. High-fidelity simulators are preferred for ensuring data quality while maintaining safety.
Measurements	Traditional ECG devices provide accurate measurements but are less practical in occupational settings. Wearable ECG devices offer convenience and applicability in real-world scenarios but require further validation.
Physiological responses	HR increases with higher MWL, though responses can vary by task and individual. HRV offers detailed insights into ANS activity but shows inconsistent results due to experimental variability.
HRV-based MWL detection system	Multi-class classification of MWL provides nuanced understanding but is challenging. Advanced deep-learning techniques and personalized models show promise for improved detection accuracy.

## Data Availability

Not applicable.
